# Dopaminergic contributions to behavioral control under threat of punishment in rats

**DOI:** 10.1007/s00213-020-05497-w

**Published:** 2020-03-27

**Authors:** Jeroen P. H. Verharen, Mieneke C. M. Luijendijk, Louk J. M. J. Vanderschuren, Roger A. H. Adan

**Affiliations:** 1 Department of Translational Neuroscience, Brain Center Rudolf Magnus, University Medical Center Utrecht, Utrecht, The Netherlands; 2 Department of Animals in Science and Society, Division of Behavioural Neuroscience, Faculty of Veterinary Medicine, Utrecht University, Utrecht, The Netherlands; 3 Department of Molecular and Cell Biology, Helen Wills Neuroscience Institute, University of California, Berkeley, Berkeley, CA 94720 USA; 4 Institute of Physiology and Neuroscience, Sahlgrenska Academy at the University of Gothenburg, Gothenburg, Sweden

**Keywords:** Rats, Dopamine, Impulsivity, Behavioral inhibition, Ventral tegmental area, Prefrontal cortex, Ventral striatum, Fiber photometry, Chemogenetics, Immediate early gene expression

## Abstract

**Rationale:**

Excessive intake of rewards, such as food and drugs, often has explicit negative consequences, including the development of obesity and addiction, respectively. Thus, choosing not to pursue reward is the result of a cost/benefit decision, proper execution of which requires inhibition of behavior. An extensive body of preclinical and clinical evidence implicates dopamine in certain forms of inhibition of behavior, but it is not fully known how it contributes to behavioral inhibition under threat of explicit punishment.

**Objectives:**

To assess the involvement of midbrain dopamine neurons and their corticostriatal output regions, the ventral striatum and prefrontal cortex, in control over behavior under threat of explicit (foot shock) punishment in rats.

**Methods:**

We used a recently developed behavioral inhibition task, which assesses the ability of rats to exert behavioral restraint at the mere sight of food reward, under threat of foot shock punishment. Using in vivo fiber photometry, chemogenetics, *c-Fos* immunohistochemistry, and behavioral pharmacology, we investigated how dopamine neurons in the ventral tegmental area, as well as its output areas, the ventral striatum and prefrontal cortex, contribute to behavior in this task.

**Results:**

Using this multidisciplinary approach, we found little evidence for a direct involvement of ascending midbrain dopamine neurons in inhibitory control over behavior under threat of punishment. For example, photometry recordings suggested that VTA DA neurons do not directly govern control over behavior in the task, as no differences were observed in neuronal population activity during successful versus unsuccessful behavioral control. In addition, chemogenetic and pharmacological manipulations of the mesocorticolimbic DA system had little or no effect on the animals’ ability to exert inhibitory control over behavior. Rather, the dopamine system appeared to have a role in the motivational components of reward pursuit.

**Conclusions:**

Together, our data provide insight into the mesocorticolimbic mechanisms behind motivated behaviors by showing a modulatory role of dopamine in the expression of cost/benefit decisions. In contrast to our expectations, dopamine did not appear to directly mediate the type of behavioral control that is tested in our task.

**Electronic supplementary material:**

The online version of this article (10.1007/s00213-020-05497-w) contains supplementary material, which is available to authorized users.

## Introduction

Inhibitory control over behavior is a process that can help to limit the pursuit of rewards like food and drugs and thereby prevent the occurrence of explicit negative consequences that are associated with its excessive intake. In humans, this may for example be the ability to limit the intake of tasty foods in order to prevent obesity, or the ability to refrain from using alcohol and drugs in order not to develop addiction (Verharen et al. 2019b). To study the process of behavioral inhibition in the face of possible punishment, we recently developed a task that studies control over the intake of sucrose pellets in rats (Verharen et al. 2019c). In this task, behavioral control is required during the presentation of an audiovisual threat signal, whereby an inability to resist eating the pellet during this threat signal is punished with a mild electric foot shock. Employing this paradigm, we showed that, after full training on the task, activity in the ventromedial region of the rat prefrontal cortex (vmPFC) is essential for the exertion of behavioral control, without any effects on task behavior when the animals could take the reward freely, i.e., without the risk of negative consequences. In contrast, the ventral striatum (VS) was important for the motivational aspects of behavior in this task (Verharen et al. 2019c).

Dopamine (DA) has been widely implicated in reward-related processes, such as incentive salience, motivation, and reward prediction, as well as in inhibitory control over behavior (Berridge 2007; Cools 2008; Pattij and Vanderschuren 2008; Dalley and Roiser 2012; Salamone and Correa 2012; Nutt et al. 2015; Schultz 2016; Verharen et al. 2019b). For example, high trait impulsivity in humans has been associated with low DA release in the striatum and low DA D2 receptor availability (Buckholtz et al. 2010; Trifilieff and Martinez 2014), and monoamine reuptake inhibitors are the first-choice treatment for impulse control disorders like attention-deficit/hyperactivity disorder (ADHD). Furthermore, functional manipulations of the DA system affect impulsive action (Cole and Robbins 1989; van Gaalen et al. 2006b; Pattij et al. 2007; Baarendse and Vanderschuren 2012; Fernando et al. 2012) and impulsive choice (Wade et al. 2000; van Gaalen et al. 2006a; Baarendse and Vanderschuren 2012; Orsini et al. 2017; Bernosky-Smith et al. 2018) in rodents, suggesting an important role of DA neurotransmission in behavioral control. However, the exact mechanism by which forebrain DA exerts control over behavior remains incompletely understood. For example, it is unknown whether DA neurons are directly engaged during the execution of behavioral control. Importantly, both the vmPFC and the VS, which play complimentary roles in performance of our behavioral inhibition task (Verharen et al. 2019c), receive dense DAergic inputs from the ventral tegmental area (VTA) (Bjorklund and Dunnett 2007; Lammel et al. 2008).

Here, we employed a multidisciplinary approach, combining behavioral pharmacology, fiber photometry, chemogenetics, and *c-Fos* immunohistochemistry to study the involvement of the mesocorticolimbic DA system in control over behavior in rats under threat of punishment. We hypothesized that VTA DA neurons directly modulate task behavior, by altering DA release in downstream regions during reward pursuit and inhibitory control. We predicted an important role of mesocortical DA in the exertion of behavioral control and of mesolimbic DA in the motivational aspects of the task, based on the phenotypes observed after pharmacological inactivation of the vmPFC and VS, respectively (Verharen et al. 2019c).

## Experimental procedures

### Animals

A total of 74 male rats with a Long-Evans background, either wild-type Rj:Orl (Janvier labs, France; for *c-Fos* and intracranial infusion experiments) or TH::Cre rats (bred in-house; for photometry and chemogenetic experiments) weighing at least 250 g at the start of the experiments, were used. Rats were housed in pairs on a 12-h/12-h reversed day-night cycle (lights off at 8 A.M.). After surgery, animals that received a head implant (for photometry and intracranial infusion experiments) were housed individually to prevent damage to the implant. All experimental procedures were conducted in agreement with Dutch laws (Wet op de Dierproeven, 2014) and European guidelines (2010/63/EU) and approved by the Animal Ethics Committee of Utrecht University and the Dutch Central Animal Testing Committee.

### Surgeries

Animals were anesthetized by an intramuscular injection of a cocktail of 0.315 mg/kg fentanyl and 10 mg/kg fluanisone (Hypnorm, Janssen Pharmaceutica, Belgium). They were then placed in a stereotaxic apparatus (David Kopf, USA), an incision was made along the midline of the skull, and craniotomies were made above the areas of interest:

VTA: AP − 5.4 mm ML  ±2.2 mm DV − 8.9 mm from skull under a 10° angle
VS: AP + 1.2 mm ML ± 2.1 mm DV − 6.3 mm from skull under a 5° angle or AP + 1.2 mm ML ± 2.7 mm DV − 7.0 mm from skull under a 10° angle
vmPFC: AP + 3.2 mm ML ± 0.6 mm DV − 3.8 mm from skull

For the VS and vmPFC, these dorsoventral coordinates reflect the position to which the guide cannulas were lowered; for the VTA, these coordinates reflect the site of viral injection.

For the intracranial infusion experiments, either one 23-G guide cannula was used that had a double protrusion, spaced 1.2 mm apart (for the vmPFC; Plastics One, USA), or two 23-G guide cannulas with a single protrusion (for the VS; Plastics One, USA) were used. Guide cannulas were lowered to the desired coordinates, secured with screws, dental glue (C&B Metabond, Parkell Prod Inc., USA), and dental cement, and the skin around the cemented cap was sutured. Dummy cannulas were placed inside the guide cannulas.

For fiber photometry, 1 μl of AAV5-FLEX-hSyn-GCaMP6s or AAV5-hSyn-eYFP (University of Pennsylvania Vector Core; 10^12^ particles/ml) was injected into the right VTA of TH::Cre rats, and an optic fiber (diameter 400 μm; Thorlabs, Germany) was lowered to 0.1-mm dorsal of the injected area and secured with screws and dental cement. For chemogenetic experiments, 0.5 μl of AAV5-hSyn-DIO-hM3Gq-mCherry (University of North Carolina Vector Core; 2 × 10^12^ particles/ml) was injected bilaterally into the VTA of TH::Cre rats. Note that these viruses have a 97% specificity for TH in this transgenic line (Boekhoudt et al. 2016a), and transfected cells may thus comprise 3% non-DAergic cells. Virus was infused at a rate of 0.2 μl/min, and the needles were kept in place for an additional 5 min after infusion to allow for diffusion of the virus into the tissue. For these experiments, measurements were conducted at least 4 weeks later to ensure proper levels of viral expression.

After surgery, all animals received carprofen for pain relief (5 mg/kg, 1×/day, for 3 days, subcutaneously) and saline for rehydration (10 ml once, subcutaneously). Animals were allowed to recover for at least a week before behavioral training started.

### Behavioral task

**Fig. 1:**
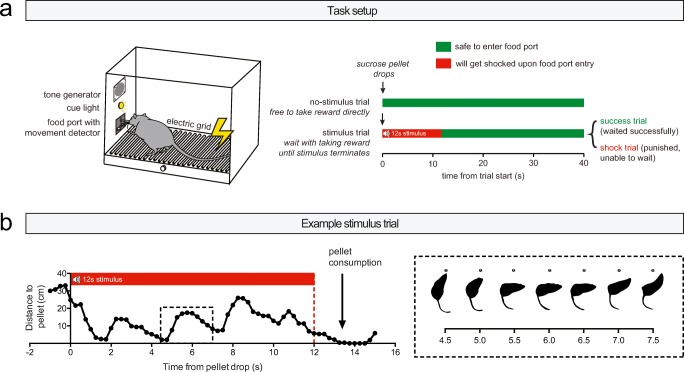
Task description. **a** Behavioral setup. Animals received 60 sucrose pellets at a fixed interval of 40 s. Half of the trials were “no-stimulus” trials, in which the animals could directly retrieve the pellet without negative consequences. The other half of the trials were “stimulus” trials, in which pellet delivery coincided with the presentation of an audiovisual threat signal. During this threat signal, food port entry was punished with an electric foot shock. **b** Quantification of behavior in an example stimulus trial, demonstrating “attract and repel” (or “approach and avoidance”) behavior towards and away from the food port during behavioral inhibition. Figure modified from Verharen et al. (2019c) with permission

The behavioral task is described in detail in Verharen et al. (2019c). In brief, we used a task that tests the ability of rats to inhibit their urge to approach a visibly present sucrose pellet during the presentation of an audiovisual threat stimulus. The task comprised 60 trials of 40 s each, in which at the start of every trial a sucrose pellet was delivered into a food port (Fig. 1a, left panel). In half of the trials, delivery of this sucrose pellet was not paired with any audiovisual cues, which signaled to the animal that it was safe to consume the pellet directly without any negative consequences (Fig. 1a, right panel, “no-stimulus trial”). In the other half of the trials, sucrose pellet delivery coincided with the onset of an audiovisual (tone + light) cue, which lasted for 12 s (Fig. 1a, right panel, “stimulus trial”). In these trials, the rat had to wait with entering the food port until stimulus termination, thus inhibiting the impulse to consume the sucrose pellet. If the rat managed to do so, it was allowed to take the pellet without further consequences (“success trial”). If the animal was not able to wait and entered the food port during the stimulus, likely reflecting a lack of control over behavior, the stimulus terminated and the animal received a mild foot shock punishment (0.3 s; “shock trial”). The intensity of this foot shock was determined for each animal separately during the training phase, but kept constant within the same animal throughout the experiment. Animals typically showed “attract and repel” (or “approach and avoidance”) behavior with regard to the sucrose pellet (Fig. 1b), indicative of behavioral conflict (Miller 1944; Verharen et al. 2019c). For the behavioral data, a shock index was computed, which represents the amount of shock trials as a fraction of the amount of shock + success trials (100 × shock/(shock + success)); i.e., it is a measure for the amount of shock trials as a function of the total stimulus trials, corrected for the number of omissions.

### Experimental procedures

Experimental procedures are described in Verharen et al. 2019c. In brief, behavioral training and testing took place during the dark phase of the reversed day-night cycle. The behavioral task was conducted in operant conditioning chambers (MedPC, Med Associates Inc., USA), equipped with a food port with an infrared movement detector, flanked by two cue lights, a pellet dispenser delivering 45-mg sucrose pellets (SP; 5TULl; TestDiet, USA), a tone generator, a house light, and a shock grid floor.

During behavioral training, animals were kept on a food restriction regimen of 4 g chow per 100 g body weight, but had ad libitum access to tap water in the home cage. Animals were trained for 5–7 days a week, and received one or two training sessions per day. In the first training phase, animals learned to retrieve a sucrose pellet that was delivered into the pellet dispenser with a fixed interval of 40 s; this was essentially the final task version but without the stimulus trials. The animals progressed to the second, final training phase when all animals retrieved the pellet in at least 55 of the 60 trials. In the final training phase, animals received the regular version of the task, and foot shock intensity was initially set at 0.35 mA. Foot shock intensity was gradually increased with 0.05 or 0.1 mA between sessions when the majority of stimulus trials was punished (punishment too mild), and was decreased if the majority of trials was omitted (punishment too intense). The foot shock intensity was kept constant for an animal when at least 20 out of 30 stimulus trials were “success” trials (final median foot shock intensity 0.50 mA; 25–75th quartile, 0.45–0.60). During behavioral testing, animals were food-restricted for ~ 3 h prior to the task.

For the photometry experiment, a slightly different version of the behavioral task was used, in which an upcoming pellet drop was signaled to the animal using the house light to ensure task engagement. In this version of the task, the house light was turned on 5 s before and turned off 30 s after a pellet drop; during the behavioral experiments, the house light was continuously on.

### Fiber photometry

**Fig. 2:**
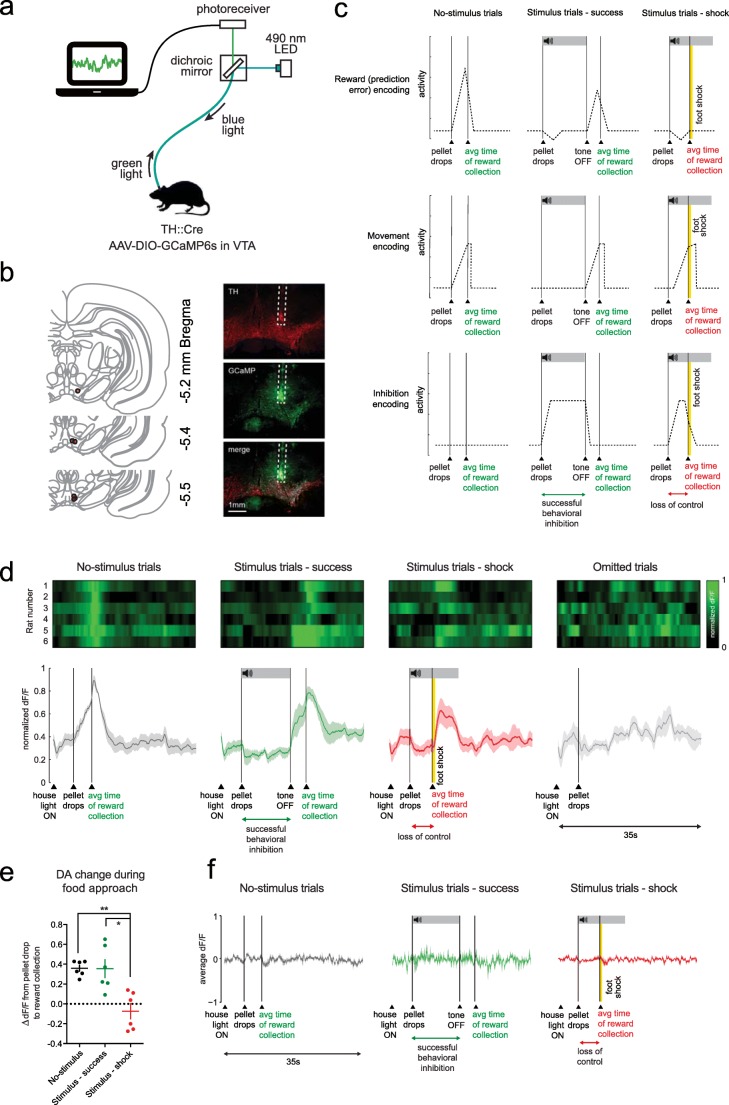
Dopamine neuron activity during behavioral inhibition. **a** Fiber photometry setup. **b** Histological verification. Red circles indicate fiber placement of individual animals. **c** Expected activity patterns. **d** VTA DA neuron normalized activity during the different trial types (*n* = 6 rats). Bottom graph shows mean ± standard error of the mean of the six animals. **e** Quantification of dF/*F* signal during food approach. **P = 0.0098, *P = 0.0105 in post hoc *t* tests (see Supplementary statistics table in Online Resource 1). **f** dF/*F* of animals injected with control fluorophore eYFP (*n* = 4 rats). Note that these graphs show the unnormalized dF/*F* (different than panel **d**)

Fiber photometry was conducted with a custom-built single wavelength fiber photometry system, as described in Verharen et al. (2018). In brief, blue 490-nm LED light (Thorlabs, USA) was lock-in amplified (Amplifier SR810; Stanford Research Systems, USA) and delivered through a patch cord (400 μm core diameter; Thorlabs, USA), connected to a stereotaxically placed optic fiber (400 μm diameter; Thorlabs, USA) using a 2.5-mm ceramic ferrule (Thorlabs, USA). Green emission light traveled back through the patch cord, was passed through a dichroic mirror (Semrock, USA), and was detected by a photodetector (Newport Corporation, USA). The signal was then passed on to the lock-in amplifier and digitized (Digidata 1550a; Molecular Devices, USA). Next, the raw signal was converted to dF/*F* values by normalizing each time point *F*_*x*_ to the baseline *F*_0_, which was defined as the average of the 50% middle values of the 30 s preceding each time point *F*_*x*_. We then re-aligned the dF/*F* traces to the average latencies of pellet retrieval of all animals, so that the different behaviors could be time-locked into one single graph, as was done in Syed et al. (2015). This was accomplished by compressing or stretching the dF/*F* signal of every trial so it would fit the average latency of pellet retrieval (the average time between pellet drop and retrieval) of the group, using the Matlab command “resizem.” Data in Fig. 2d were normalized to the 0–1 range to correct for inter-animal variation in signal strength; this was done by setting the lowest average dF/*F* value for that animal across the four trial types to 0, and the highest to 1: *F*_*x*,norm_ = (*F*_*x*_ − *F*_min_)/(*F*_max_ − *F*_min_).

### Chemogenetics

Animals were injected i.p. with the hM3Dq ligand clozapine-N-oxide (CNO; NIH Drug Supply Program) at a dose of 0.5 mg/kg dissolved in saline. After injection, animals were placed back into their home cage for 20–30 min, before behavioral testing commenced. For the locomotor test, animals were injected with CNO 10 min after the start of the experiment (denoted by an arrow in the graph).

### Intracranial infusions

For the infusion experiments, injectors were used that protruded 0.6 (VS) or 1 (vmPFC) mm beyond the termination point of the guide cannulas. One day before the infusions, animals were habituated to the infusion procedure by infusion of 0.3 μl sterile saline. On testing day, animals received an infusion of 0.3 μl saline or 20 μg of cis-(Z)-α-flupenthixol dihydrochloride (Sigma-Aldrich, The Netherlands) dissolved in 0.3 μl saline (counterbalanced between days). For the vmPFC inactivation experiment, 0.3 μl saline or 0.3 μl saline containing baclofen (1 nmol; Sigma-Aldrich, Netherlands) and muscimol (0.1 nmol; Sigma-Aldrich, Netherlands) was used. The drugs were infused with an infusion pump (Harvard Apparatus, USA), set at a rate of 0.5 μl/min. After infusion, injectors were kept in place for an additional 30 s (to allow for diffusion of the drug into the tissue), and the animals were placed back in the home cage for 10–20 min before testing began.

### *c-Fos* immunohistochemistry

For the *c-Fos* experiments, 18 animals were trained on the normal version of the task with 60 trials, comprising 30 stimulus trials and 30 no-stimulus trials. During the test session, half of the group received 25 stimulus trials (“stimulus group”) and the other half received 25 no-stimulus trials (“no-stimulus group”). In a previous experiment, we omitted the foot shocks from the stimulus trials (to prevent the foot shocks themselves from inducing *c-Fos* expression); however, this directly leads to a dramatic reduction in the number of successfully waited trials (i.e., fast extinction of the inhibition response), so we decided to include foot shock in the stimulus group (if animals retrieved the pellet during the stimulus).

Ninety minutes after termination of the behavioral task, the animals were transcardially perfused with phosphate-buffered saline (PBS) followed by 4% paraformaldehyde (PFA) in PBS. Brains were post-fixed in PFA for 24 h at 4 °C followed by a 30% sucrose solution at 4 °C. Brain sections (40 μm) were cut on a cryostat and were stained for *c-Fos* using a 3,3′-diaminobenzidine (DAB) protocol. First, the sections were blocked for 60 min at room temperature using a mixture of 10% normal goat serum and 0.5% Triton-X in PBS and were then incubated in primary rabbit antibody directed against *c-Fos* (1:1000; Cell Signaling) in 3% normal goat serum in PBS, overnight at room temperature. The next day, the sections were washed with PBS and incubated with a secondary biotinylated goat antibody directed against rabbit (1:200; Vector labs) for 120 min in 3% normal goat serum at room temperature. Sections were then washed in PBS and incubated in biotin/avidin (1:1000; Vectastain) in PBS for 60 min. Afterwards, the sections were stained for 3 min using liquid DAB (Dako) with 2% nickel ammonium sulfate. After staining, the sections were dehydrated and mounted with a xylene-based mounting medium.

Sections (approximately + 1.5 mm Bregma for VS and + 3.7–4.2 mm Bregma for vmPFC) were photographed using a brightfield microscope (at a × 5 magnification; AxioImager M2), and *c-Fos* analysis was performed in a semi-automated fashion using an ImageJ (Version 1.51s) routine (Verharen et al. 2019a). First, the microscopic images were Fourier-transformed, and a band-pass filter was applied, band-pass filtering structures of approximately the size of *c-Fos*-expressing nuclei (filter was set between 3 and 6 pixels). Next, peaks in the band-passed image were found using ImageJ’s “Find maxima” function (threshold was set at 145). For each region of interest, the total number of *c-Fos-*expressing cells and the surface area were calculated, which were used to compute the density of *c-Fos* in that region of interest.

### Histological verification

After the behavioral experiments, animals were transcardially perfused, and brains were sliced according to the protocol described above in paragraph “*c-Fos* immunohistochemistry.” For chemogenetic experiments, VTA sections (50 μm) were cut on a cryostat and stained for hM3Dq and tyrosine hydroxylase (TH) by using free-floating immunohistochemistry. First, sections were blocked for 60 min using 3% normal goat serum and 0.3% Triton-X in PBS, and then incubated overnight at 4 °C using primary antibodies (1:1000) directed against mCherry (rabbit anti-dsRed; Clontech Living Colors #632496) and TH (mouse anti-TH; Millipore #MAB318) in blocking solution. The next day, sections were washed in PBS and incubated for 120 min with secondary antibodies (1:1000) against rabbit (goat anti-rabbit 568; Abcam #175471) and mouse (goat anti-mouse 488; Abcam #150113). Brain slices were then mounted and coverslipped using FluorSave (Merck Millipore, USA). Images were photographed using an epimicroscope to ensure bilateral expression of the hM3Dq-mCherry. For histological verification of the infusion sites, brain sections were mounted and colored with 5% Giemsa (Sigma-Aldrich, The Netherlands) dissolved in distilled water.

### Exclusion criteria

Histological verification of infusion sites and viral expression was performed by an experimenter blind to the experimental results. One animal from the vmPFC infusion group was excluded based on misplacement of the cannulas. One animal from the VS infusion group was excluded because it lost its head cap. Four animals were excluded from the VTA hM3Dq group because of unilateral expression (2 animals), no expression (1 animal), or hydrocephalus (1 animal). Two animals were excluded from the *c-Fos* experiment: one animal because it was hydrocephalic and one animal because the *c-Fos* staining had not worked (presumably because of an experimental mistake during the staining process). Infusions in the VS were initially separately targeted at the nucleus accumbens shell and core, given their differential involvement in aversive behaviors (Piantadosi et al. 2018), but these groups were eventually combined because the areas were difficult to histologically distinguish, and it was unclear whether the infused volume remained restricted to these NAc subregions.

### Code availability

The MedPC script of the task is available at http://www.github.com/jeroenphv.

### Statistics

Statistical tests were performed with Prism 6.0 (GraphPad Software Inc., USA). For the dF/*F* response to food approach of the photometry experiment, a one-way repeated measures of analysis of variance (ANOVA) was used, with stimulus type as a within-subjects repeated measures factor, followed by a Bonferroni post hoc test when appropriate. For the locomotor test, a two-way repeated measures ANOVA was used in the time-bin analysis (with time-bin as a within-subjects repeated measures factor and genotype as a between-subjects factor) and an unpaired *t* test in the cumulative distance moved analysis. For the data of the behavioral control task, individual paired *t* tests were used to compare treatment (CNO or α-flupenthixol) with baseline (saline). For the *c-Fos* experiment, a two-way ANOVA was used with brain area and group as between-subject factors. In all figures, the statistical range is denoted as follows: *P < 0.05, **P < 0.01, ***P < 0.001, ****P < 0.0001. All test statistics are presented in the Supplementary statistics table in Online Resource 1.

## Results

### VTA DA neurons do not encode control over behavior

To study the activity of midbrain DA neurons during successful and unsuccessful control over behavior, we measured population activity of medial VTA DA neuron cell bodies by employing in vivo fiber photometry (Gunaydin et al. 2014) in TH::Cre rats (Fig. 2a, b). Based on the different theories of DA function, we formulated three hypotheses about the expected activity patterns (Fig. 2c). First, DA neurons may encode reward or reward prediction errors (Schultz et al. 1997; Schultz 2016), resulting in increased activity when animals can retrieve the pellet without punishment (i.e., after pellet drop in no-stimulus trials or after tone offset in stimulus trials) and possibly reduced DA neuron activity during stimulus onset (if animals experience the threat cue as aversive). Second, we hypothesized that neurons encode movement towards the pellet (Howe and Dombeck 2016). Third, neurons may directly encode inhibition of behavior (Mazzoni et al. 2007; Syed et al. 2015).

To be able to make a direct comparison between the different animals and trial types, we re-aligned the neuronal population activity to the average response latencies of the animals, by compressing or stretching the dF/*F* signal (see ref. Syed et al. 2015). This analysis revealed a neuronal activation pattern (Fig. 2d) that is reminiscent of the pattern expected based on the encoding of reward or reward prediction errors (Fig. 2c, top panel). Thus, during “no-stimulus” trials, in which the animals were free to take the sucrose pellet directly without negative consequences, we observed a ramping of DA neuron activity from pellet presentation to retrieval, with a decline in activity back to baseline afterwards. Similarly, during “stimulus—success” trials, in which animals showed successful control over behavior, we observed this same ramping after tone offset, i.e., when animals were free to take the pellet without negative consequences. No obvious changes in DA neuron activity were observed during successful behavioral control. During “Stimulus—shock” trials, in which animals retrieved the pellet during stimulus presentation and thus received foot shock punishment, we observed a similar response during the inhibition period as during “stimulus—success” trials, i.e., no changes in DA neuron activity during stimulus presentation. We observed an increase in DA neuron activity after foot shock delivery, which is something we have observed before (Verharen et al. 2018) and perhaps reflects the salience of or relief from the shock. Finally, omitted trials, in which the animals did not retrieve the food pellet during the entire 40-s trial period, did not evoke any detectable changes in DA neuron activity. Comparing the changes in dF/*F* value during approach to the sucrose pellet (Fig. 2e) demonstrated higher dF/*F* responses during food approach in “no-stimulus” and “stimulus—success” trials as compared with “stimulus—shock” trials, suggesting that these DA neurons did not merely encode reward-directed movement. Importantly, no changes in fluorescence were observed in animals that were injected with a control fluorophore (Fig. 2f). In sum, these data suggest that VTA DA neurons encode value-related signals, but not (successful or unsuccessful) behavioral control or movement.

### VTA DA neuron activation does not affect task performance

**Fig. 3:**
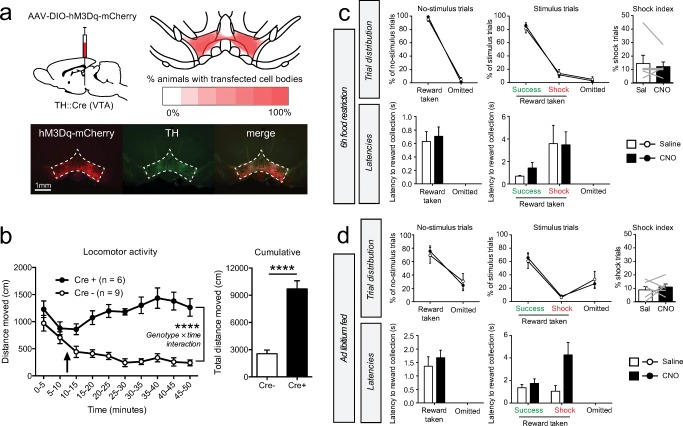
Chemogenetic VTA DA neuron activation. **a** Experimental procedure and histological verification of hM3Dq-mCherry expression. Bottom and top right images represent coronal slices of the VTA. **b** Locomotor test. Arrow indicates i.p. CNO injection (0.5 mg/kg). ****P < 0.0001 (see Supplementary statistics table in Online Resource 1). **c** Task performance after i.p. CNO injection in hungry animals (*n* = 6 rats). The shock index is a measure for the relative amount of shock trials compared for the number of omissions and is computed by 100% × shock trials/(shock + success trials). Gray lines in the shock index graphs indicate individual animals. **d** Task performance after CNO injection in ad libitum fed animals (*n* = 6 rats). Gray lines in the shock index graphs indicate individual animals

To assess whether hyperactivity of these same VTA DA neurons hampers the exertion of behavioral control, we injected TH::Cre rats with a viral vector expressing the excitatory chemogenetic receptor hM3Dq fused to mCherry-fluorescent protein bilaterally into the VTA (Fig. 3a), leading to expression in the entire VTA (projecting to vmPFC and the VS). To confirm functional activation of these neurons, we assessed locomotor activity (Boekhoudt et al. 2016a) after injection of the hM3Dq ligand clozapine-N-oxide (CNO) and observed an increase in the distance traveled in animals that were TH::Cre positive as compared with TH::Cre negative animals (Fig. 3b).

Contrary to our expectations, we observed no effects of chemogenetic VTA DA neuron activation on task performance in food-restricted animals (Fig. 3c). Given that food restriction increases baseline firing of DA neurons (Hommel et al. 2006; Branch et al. 2013), we speculated that firing in these neurons could already have been high before CNO injection, and this may have therefore masked an effect of VTA DA neuron stimulation on task behavior. Therefore, we repeated the experiment in ad libitum fed animals, but we again observed no effects of neuronal activation on task performance (Fig. 3d). These findings indicate that increasing the activity of VTA DA neurons does not alter the ability of animals to exert inhibitory control over behavior.

### Stimulus trials engage the VS, but not vmPFC

**Fig. 4:**
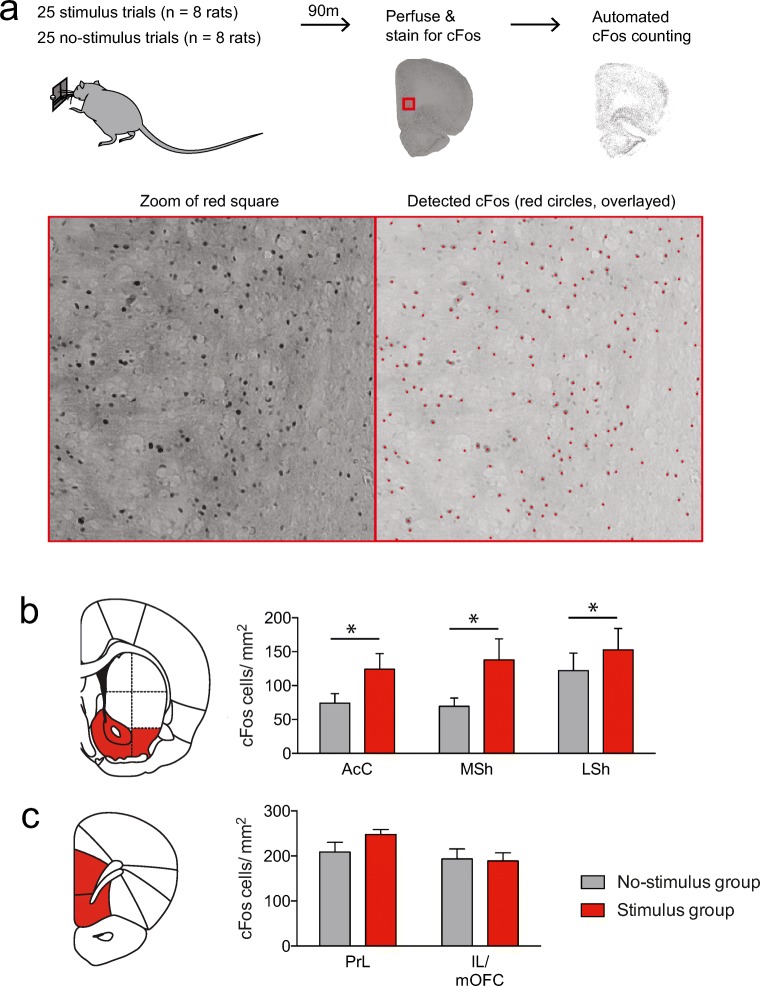
Animals that received stimulus trials showed enhanced *c-Fos* expression in the VS, but not vmPFC, as compared with animals that received no-stimulus trials. **a** Experimental procedure. Red dots in bottom right figure show the detection of *c-Fos* by the algorithm. **b***c-Fos* density was enriched across the entire VS after stimulus trials (*P = 0.0153, main effect of group in ANOVA; see Supplementary statistics table in Online Resource 1). AcC, nucleus accumbens core; MSh, medial shell of the nucleus accumbens; LSh, lateral shell of the nucleus accumbens. **c** Stimulus trials did not evoke changes in *c-Fos* density in the vmPFC (See Supplementary statistics table in Online Resource 1). PrL, prelimbic cortex; IL, infralimbic cortex; mOFC, medial orbitofrontal cortex

To explore whether the two major VTA DA output regions, the VS and vmPFC, are recruited during stimulus trials, we tested a group of animals in a task version that comprised exclusively no-stimulus trials and a different group of animals in a task version that comprised exclusively stimulus trials, and stained the brain sections for the immediate early gene *c-Fos*, as a proxy for neuronal activity (Bullitt 1990; Morgan and Curran 1991) (Fig. 4a). We then performed semi-automated cell counting on two coronal slices that included the vmPFC and VS, and compared the cumulative density of *c-Fos* levels in these brain regions.

A two-way analysis of variance (ANOVA) on the *c-Fos* density in the three major subregions of the VS revealed a significant main effect of group, but no group × brain region interaction effect, indicating that *c-Fos* expression was increased across the entire VS during stimulus trials (Fig. 4b). In contrast, no effects of group or a group × brain region interaction effect were found for *c-Fos* density in the vmPFC (Fig. 4c). No significant correlation was observed between *c-Fos* expression and the number of shocks the animals in the stimulus group received (Online Resource 2). Together, these findings suggest that the VS, but not the vmPFC, is recruited during stimulus trials.

### Blockade of DA receptors in the VS and vmPFC affects task performance

**Fig. 5:**
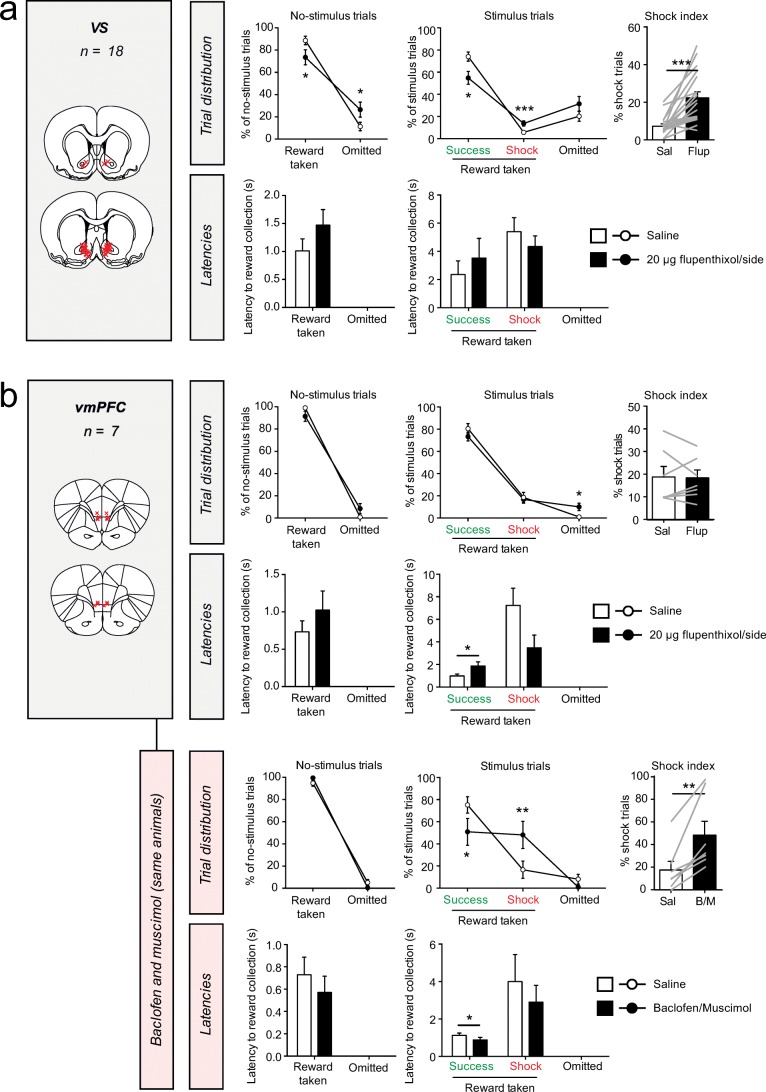
Effects of infusion of DA receptor antagonist α-flupenthixol in DA target regions. DA receptor blockade by intra-VS (**a**) or intra-vmPFC (**b**, top panel) infusion of α-flupenthixol had differential effects on task performance. Pharmacological inactivation of the vmPFC did evoke loss of behavioral inhibition (**b**, bottom panel). Red crosses in the coronal brain sections represent the infusion sites in each experiment. Gray lines in the shock index graphs indicate individual animals. ***P < 0.001, *P < 0.05 in paired *t* test (see Supplementary statistics table in Online Resource 1)

To investigate the importance of DAergic neurotransmission in VTA target regions for performance in the task, we tested the effects of infusion of the DA receptor antagonist α-flupenthixol into the VS and vmPFC (Fig. 5). During no-stimulus trials, we observed a significant increase in the number of omissions after α-flupenthixol infusion into the VS (Fig. 5a). In stimulus trials, we observed a significant decrease in the number of success trials and a significant increase in the number of shock trials, but no significant effect on the number of omissions. Hence, the shock index was significantly increased after α-flupenthixol infusion. No effects were observed on the latency of pellet retrieval in either trials.

Infusion of α-flupenthixol into the vmPFC had no effects on behavior during no-stimulus trials (Fig. 5b, top panel). During stimulus trials, it resulted in a significant (yet numerically modest) increase in the number of omissions, but no significant changes in the number of success or shock trials. We further observed a significant increase in the latency of pellet retrieval in success trials, but not in shock trials. To confirm that we targeted the same vmPFC region that was recently implicated in control over behavior in this task (Verharen et al. 2019c), we used the same animals and infused the GABA receptor agonists baclofen and muscimol into this region, which induces a transient inactivation of the vmPFC. We observed that this manipulation indeed impaired inhibition of behavior, i.e., a reduction in the number of success trials and an increase in shocks received during stimulus trials, but no changes in behavior during no-stimulus trials. This replicates our previous findings (Verharen et al. 2019c).

## Discussion

In this study, we have utilized a recently developed task to assess the contribution of the mesocorticolimbic DA system to inhibitory control over behavior in rats. By combining this task with in vivo fiber photometry, chemogenetics, *c-Fos* immunohistochemistry, and behavioral pharmacology, we have provided novel insights into the role of DA in behavioral control and other aspects of task performance. We show that VTA DA neurons are not engaged during behavioral control and that activation of these neurons does not affect the animals’ ability to exert behavioral restraint. Blockade of VS DA receptors mildly reduced behavioral control and task engagement, whereas vmPFC DA receptor antagonism hardly altered task performance. Together, our data demonstrate an involvement of ascending DA neurons and their forebrain efferent regions in aspects of task performance related to reward prediction and incentive motivation, rather than behavioral inhibition.

First, we have visualized neuronal dynamics in vivo when animals displayed successful and unsuccessful control over behavior by employing fiber photometry in TH::Cre rats. The photometry fibers were for the most part placed in the medial aspect of the paranigral nucleus of the VTA. As a result, our measurements were mostly from DA neurons projecting to the medial part of the VS and the vmPFC (Lammel et al. 2008; Lammel et al. 2014; Morales and Margolis 2017), which are the regions we also targeted in our subsequent intracranial infusion experiments. Of our three hypotheses (Fig. 2c), VTA DA neuron activity most closely represented a pattern of coding of reward or reward prediction errors, rather than movement or behavioral inhibition. That said, on the basis of the present observations, we cannot strictly rule out that VTA DA neuron activity encoded incentive salience, as it is difficult to predict whether and how the threat cue and the foot shocks would be perceived as salient, and how these would influence the salience of reward presentation in the current behavioral paradigm. Importantly though, the neuronal responses are unlikely to reflect movement, given the lack of transients during food port approach in shock trials (Fig. 2d, e). This observation is in line with a recent finding that actions are encoded differently by VTA DA neurons depending on whether there is a risk of punishment or not (Park and Moghaddam 2017).

One surprising observation of the photometry experiments was that during threat cue presentation, neuronal activity was similar in success and shock trials, suggesting that VTA DA neurons do not directly govern control over behavior. In fact, the threat cue itself did not elicit strong changes in neural activity, neither upwards nor downwards. Whether this lack of any distinct neural activity patterns during the threat cue is in line with these neurons encoding value-related signals is subject to debate. For example, one may argue to expect (1) downward, negative prediction error–like, transients, (2) upward transients, signaling the receipt of sucrose, or (3) a suppression of a pellet delivery-induced positive transient by the threat cue, for example, because of counterconditioning (Dickinson and Pearce 1977) or due to the cue acting as a discriminative stimulus or “occasion setter” (Fraser and Holland 2019). In addition, in well-trained animals, the threat cue does not have to be aversive per se, as it may merely predict upcoming (delayed) reward and not punishment (which can be prevented by waiting). Moreover, since foot shock intensity was slowly increased during training, the punishment itself may have become less aversive through adaptation (Solomon 1964). Important to note is that the “attract and repel” behavior (Fig. 1b) that we typically observed during presentation of the threat cue is thought to represent internal conflict, which has been theoretically and experimentally delineated by Miller (1944). This behavior, also referred to as “approach and avoidance”, is thought to arise from oscillatory behavior around a spatial equilibrium in which the pull of the reward is equal to the drive to avoid the punishment (Miller 1944). In accordance with this theory, during these oscillations, we observed quicker retreat from the reward than approach towards it (Fig. 1b). An interesting follow-up experiment would be to study whether neurons in the DA system can be found that encode the switch from approach towards avoidance behavior (and vice versa) during behavioral inhibition using frame-by-frame video analysis of task behavior.

Despite observing general hyperactivity after chemogenetic activation of VTA DA neurons (covering the entire VTA; projecting to vmPFC and the entire VS), we did not observe any behavioral changes in the task, supporting the notion that these VTA DA neurons do not directly modulate control over behavior. This finding was somewhat surprising to us given the broad role that VTA DA has on many aspects of reward-related and motivated behaviors (Robbins and Everitt 2007; Salamone and Correa 2012) and impulsivity (Pattij and Vanderschuren 2008; Eagle and Baunez 2010; Dalley and Robbins 2017). Our photometry data may, however, provide clues about the reason for the lack of effect of DA neuron stimulation on task behavior: we have previously shown (Verharen et al. 2018) that DA neuron stimulation primarily affects processing of negative RPE-like signals in the accumbens (resulting in loss and punishment insensitivity). The fact that we did not see any clear sub-baseline transients in our photometry experiment may indicate that there were no dips in DA neuron activity that the chemogenetic activation could interfere with. We speculate that neuronal hyperactivation may merely amplify the upward transients that were already present during certain moments in the task, and as a result did not affect task performance. In line with this notion is that the experiments were performed after the animals had reached stable performance in the task, so that little to no learning was involved during testing. DA is thought to be an important mediator of value-based learning processes (Schultz 2016), and a mere disruption of learning would not affect behavior in this stage of task performance.

Consistent with the lack of effect of DA neuron stimulation on our task is a recent study from our lab that showed that VTA DA neuron activation in rats did not increase motor impulsivity in the 5-choice serial reaction time task (Boekhoudt et al. 2016b). This suggests that the lack of effects of DA neuron stimulation on behavioral control extends towards other forms of impulsivity, although this is in sharp contrast with previous studies on impulsivity using DA receptor agonists (Pattij and Vanderschuren 2008; Fernando et al. 2012). Therefore, DA may not be strongly involved in the type of behavioral inhibition that is assessed in our task—that is, behavioral inhibition under direct threat of (foot shock) punishment, as long as there is no learning involved.

The *c-Fos* experiment showed increased neuronal activation in the entire VS, but not the vmPFC, in animals that exclusively received stimulus trials compared with animals that only received no-stimulus trials in the task. This suggests that the VS is actively engaged during stimulus trials. On the basis of our previous pharmacological inactivation of the VS (Verharen et al. 2019c), and the fiber photometry and chemogenetics experiments in the present study, we think that it is unlikely that the increase in *c-Fos* expression reflects the VS mediating behavioral control. Rather, it may be the result of other aspects of stimulus trials, like the threat of foot shock (Beck and Fibiger 1995), which is difficult to completely control for, and our data should therefore be interpreted with caution. Furthermore, it is interesting to note that we did not observe any significant changes in *c-Fos* expression in the vmPFC, given that we have previously demonstrated impairments in behavioral control after pharmacological inactivation of this region (Verharen et al. 2019c). That said, the exertion of behavioral control by the vmPFC does not necessarily have to be the result of a general increase in the region’s activity, but may as well be due to more subtle changes in activity, such as alterations in the neural computations within the vmPFC or of changed activity in a subpopulation of vmPFC neurons.

DA receptor blockade in the NAc significantly decreased the number of success trials and increased the absolute number of shock trials. Although this effect was numerically more modest than the phenotype observed after pharmacological inactivation of the vmPFC (Verharen et al. 2019c), it does suggest decreased inhibitory control. However, during no-stimulus trials, which can be seen as a control to detect any general impairments in behavior, an increase in omissions was observed. Therefore, the effects of NAc DA receptor antagonism on behavioral control should be interpreted with caution, not least since the fiber photometry and chemogenetic experiments yielded no evidence for an important role for DA in behavioral inhibition in this task. Thus, the observed pattern of effects after DA receptor antagonist infusion may not primarily have been driven by changes in behavioral control, but rather by the disruption of other cognitive processes. For example, it could be the case that DA released during unpunished reward delivery, as we have shown with our photometry experiment, cannot be detected by DA receptors in the NAc, which may lead to alterations in motivation or impairments in the detection of pellet delivery and stimulus presentation.

Finally, pharmacological blockade of DA receptors in the vmPFC did alter behavior during stimulus trials, without affecting behavior during no-stimulus trials. Interestingly, this did not seem to be related to behavioral control, but rather by a decreased motivation for reward in stimulus trials. As such, we observed an increase in the number of omissions and an increased latency of pellet retrieval during success trials. This phenotype is different than the one induced by pharmacological inactivation of the vmPFC, which was characterized by impairments in inhibitory control (i.e., an increase in the number of shock trials, without effects on the number of omissions or on behavior in no-stimulus trials), indicating that the role of the vmPFC in inhibitory control is not governed by DAergic neurotransmission. Instead, the subtle effects of intra-vmPFC flupenthixol infusion on task behavior might be related to the role of mesocortical DA in weighing the costs and benefits of different courses of actions (Floresco 2013; van Holstein and Floresco 2019), especially since the effects were restricted to stimulus trials, in which animals actively need to weigh the negative consequences of reward pursuit against the positive effects of sucrose ingestion.

In sum, we have used a multidisciplinary approach to test the hypothesis that mesocorticolimbic DA is involved in the exertion of inhibitory control over food intake under threat of punishment. We found little evidence in support of this hypothesis, as we did not observe changes in VTA DA neuron activity during successful and unsuccessful behavioral control. Furthermore, chemogenetic DA neuron activation did not affect task performance. We did find increased *c-Fos* expression in the VS during stimulus trials, and DA receptor blockade within the VS resulted in a modest increase in the amount of shock trials, but this may not necessarily have been the result of a direct impairment in the ability to exert behavioral control. Furthermore, DA receptor blockade in the vmPFC did not change measures of inhibitory control, even though we have previously shown that activity in this area is essential for this behavior (Verharen et al. 2019c). Together, our findings contribute to the understanding of the role of DA in motivated behaviors, by showing a modulatory role of mesocorticolimbic DA in the expression of cost/benefit decisions.

## Electronic supplementary material

ESM 1 (PDF 481 kb)
