# Supplementary material for: Dopaminergic contributions to behavioral control under threat of punishment in rats
Source: Psychopharmacology (Berl). 2020 Mar 27;237(6):1769–82. doi: 10.1007/s00213-020-05497-w (PMC7239833; doi:10.1007/s00213-020-05497-w)
Supplement: Supplementary file 1 — (PDF 481 kb) [file 213_2020_5497_MOESM1_ESM.pdf]

Online Resources

Dopaminergic contributions to behavioral control under threat of punishment in rats

Jeroen P.H. Verharen[1,2,3], Mieneke C.M. Luijendijk[1], Louk J.M.J. Vanderschuren[2,5], Roger A.H. Adan[1,4,5,*]

[1] Brain Center Rudolf Magnus, Department of Translational Neuroscience, University Medical Center Utrecht, Utrecht, The Netherlands.
[2] Department of Animals in Science and Society, Division of Behavioural Neuroscience, Faculty of Veterinary Medicine, Utrecht University, Utrecht, The Netherlands.
[3] Helen Wills Neuroscience Institute, Department of Molecular and Cell Biology, University of California Berkeley, Berkeley, CA 94720, United States
[4] Institute of Physiology and Neuroscience, Sahlgrenska Academy at the University of Gothenburg, Gothenburg, Sweden.
[5] These authors contributed equally.

* Corresponding author: r.a.h.adan@umcutrecht.nl, +31-88-7568810

Online Resource 1
Supplementary statistics table

Figure	Parameter	n	Test	Test statistic	P value	Significance
2e	dF/F change	6 rats	1-way repeated measures ANOVA	F (1.722, 8.609) = 14.69	p = 0.0021	**
	No stim vs Stim-Success	6 rats	Paired t-test, Bonferroni correction	t(5) = 0.3438	p > 0.999	
	No stim vs Stim-Shock	6 rats	Paired t-test, Bonferroni correction	t(5) = 5.190	p = 0.0105	*
	Stim-Succes vs Stim-Shock	6 rats	Paired t-test, Bonferroni correction	t(5) = 5.271	p = 0.0098	**
3b, left panel	Whole graph	6 rats (Cre+)<br>9 rats (Cre-)	2-way repeated measures ANOVA	Main time effect: F(9,117) = 3.538<br><br>Main effect genotype: F(1,13) = 47.26<br><br>Genotype * time interaction: F(9,117) = 9.802	All p < 0.0001	****
3b, right panel	Whole graph	6 rats (Cre+)<br>9 rats (Cre-)	Unpaired t-test	t(13) = 8.162	p < 0.0001	****
3c	Stim - Reward taken	6 rats	Paired t-test	t(5) = 1.151	p = 0.3019	
	Stim - Omitted	6 rats	Paired t-test	t(5) = 1.151	p = 0.3019	
	NoStim - Success	6 rats	Paired t-test	t(5) = 0.7906	p = 0.4650	
	NoStim - Shock	6 rats	Paired t-test	t(5) = 0.6984	p = 0.5160	
	NoStim - Omitted	6 rats	Paired t-test	t(5) = 0.5096	p = 0.6320	
	Shock index	6 rats	Paired t-test	t(5) = 0.7130	p = 0.5077	
	Latency NoStim	6 rats	Paired t-test	t(5) = 1.387	p = 0.2240	
	Latency Stim - Success	6 rats	Paired t-test	t(5) = 1.550	p = 0.1819	
	Latency Stim - Shock	6 rats	Paired t-test	t(5) = 0.0525	p = 0.9602	
3d	Stim - Reward taken	6 rats	Paired t-test	t(5) = 0.4218	p = 0.6907	
	Stim - Omitted	6 rats	Paired t-test	t(5) = 0.4218	p = 0.6907	
	NoStim - Success	6 rats	Paired t-test	t(5) = 0.4021	p = 0.7042	
	NoStim - Shock	6 rats	Paired t-test	t(5) = 0.8885	p = 0.4150	

Figure	Parameter	n	Test	Test statistic	P value	Significance
	NoStim - Omitted	6 rats	Paired t-test	t(5) = 0.5505	p = 0.6057	
	Shock index	6 rats	Paired t-test	t(5) = 0.6041	p = 0.5721	
	Latency NoStim	6 rats	Paired t-test	t(5) = 1.314	p = 0.2460	
	Latency Stim - Success	6 rats	Paired t-test	t(5) = 0.9701	p = 0.3766	
	Latency Stim - Shock	5 rats	Paired t-test	t(4) = 2.651	p = 0.0569	
		One animal had no Stim-Shock trials after saline injection, so was excluded from statistical comparison				
4b	c-Fos density NAc	8 vs 8 rats	Two-way ANOVA	Main effect group F(1,42) = 6.40<br><br>Main effect brain regions F(2,42) = 1.51<br><br>Group * brain region interaction effect F(2,42) = 0.31	Main effect group p = 0.0153<br><br>Main effect brain regions p = 0.2332<br><br>Group * brain region interaction effect p = 0.7353	*
4c	c-Fos density vmPFC	8 vs 7 rats	Two-way ANOVA	Main effect group F(1,26) = 0.91<br><br>Main effect brain regions F(1,26) = 4.17<br><br>Group * brain region interaction effect F(1,26) = 1.38/	Main effect group p = 0.3500<br><br>Main effect brain regions p = 0.0515<br><br>Group * brain region interaction effect p = 0.2507	
		For one animal from the no-stimulus group, no PFC slices could be retrieved.				
5a	Stim - Reward taken	18 rats	Paired t-test	t(17) = 2.601	p = 0.0186	*
	Stim - Omitted	18 rats	Paired t-test	t(17) = 2.601	p = 0.0186	*
	NoStim - Success	18 rats	Paired t-test	t(17) = 2.857	p = 0.0109	*
	NoStim - Shock	18 rats	Paired t-test	t(17) = 4.448	p = 0.0004	***
	NoStim - Omitted	18 rats	Paired t-test	t(17) = 1.643	p = 0.1188	
	Shock index	18 rats	Paired t-test	t(17) = 5.003	p = 0.0001	***
	Latency NoStim	18 rats	Paired t-test	t(17) = 1.439	p = 0.1683	
	Latency Stim - Success	18 rats	Paired t-test	t(17) = 0.7419	p = 0.4683	
	Latency Stim - Shock	16 rats	Paired t-test	t(15) = 0.5158	p = 0.6135	
		Two animals had no Stim-Shock trials after saline infusion, so were excluded from statistical comparison				
5b (top)	Stim - Reward taken	7 rats	Paired t-test	t(6) = 2.027	p = 0.0891	

Figure	Parameter	n	Test	Test statistic	P value	Significance
	Stim - Omitted	7 rats	Paired t-test	t(6) = 2.027	p = 0.0891	
	NoStim - Success	7 rats	Paired t-test	t(6) = 1.457	p = 0.1954	
	NoStim - Shock	7 rats	Paired t-test	t(6) = 0.7947	p = 0.4571	
	NoStim - Omitted	7 rats	Paired t-test	t(6) = 2.502	p = 0.0464	*
	Shock index	7 rats	Paired t-test	t(6) = 0.1675	p = 0.8725	
	Latency NoStim	7 rats	Paired t-test	t(6) = 2.435	p = 0.0508	
	Latency Stim - Success	7 rats	Paired t-test	t(6) = 2.627	p = 0.0392	*
	Latency Stim - Shock	7 rats	Paired t-test	t(6) = 2.443	p = 0.0503	
5b (bottom)	Stim - Reward taken	7 rats	Paired t-test	t(6) = 2.085	p = 0.0821	
	Stim - Omitted	7 rats	Paired t-test	t(6) = 2.085	p = 0.0821	
	NoStim - Success	7 rats	Paired t-test	t(6) = 2.540	p = 0.0441	*
	NoStim - Shock	7 rats	Paired t-test	t(6) = 4.368	p = 0.0047	**
	NoStim - Omitted	7 rats	Paired t-test	t(6) = 1.947	p = 0.0994	
	Shock index	7 rats	Paired t-test	t(6) = 4.208	p = 0.0056	**
	Latency NoStim	7 rats	Paired t-test	t(6) = 0.7745	p = 0.4681	
	Latency Stim - Success	7 rats	Paired t-test	t(6) = 2.460	p = 0.0491	*
	Latency Stim - Shock	6 rats	Paired t-test	t(5) = 0.3608	p = 0.7330	
		One animal had no Stim-Shock trials after saline infusion, so was excluded from statistical comparison				

Online Resource 2
Supplementary to Figure 4
cFos expression versus shocks received

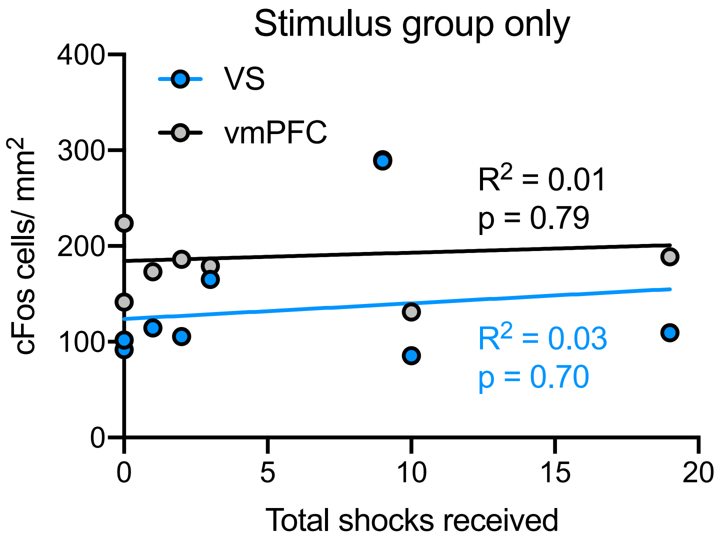

The number of shocks the animals in the stimulus group received did not correlate with *cFos* density in VS or vmPFC.
